# Tucum-do-cerrado (*Bactris setosa* Mart.) modulates oxidative stress, inflammation, and apoptosis-related proteins in rats treated with azoxymethane

**DOI:** 10.1371/journal.pone.0206670

**Published:** 2018-11-14

**Authors:** Natália A. Campos, Marcela S. B. da Cunha, Sandra F. Arruda

**Affiliations:** 1 Postgraduate Program in Human Nutrition, Faculty of Health Sciences, Campus Universitário Darcy Ribeiro, Universidade de Brasília, Brasília, Brazil; 2 Biological and Health Sciences Center, Campus Reitor Edgard Santos, Universidade Federal do Oeste da Bahia, Barreiras, Bahia, Brazil; 3 Department of Nutrition, Faculty of Health Sciences; Campus Universitário Darcy Ribeiro, Universidade de Brasília, Brasília, Distrito Federal, Brazil; Inserm U0152, UMR 5286, FRANCE

## Abstract

Oxidative and inflammatory responses play an important role in the development and prevention of cancer, with both responses being modulated by phytochemical compounds. This study investigated the chemopreventive effect of tucum-do-cerrado fruit in rats treated with azoxymethane. Wistar rats were treated for 12 weeks with: a control diet (CT); a control diet + AOM (CT/DR); a control diet + 15% tucum-do-cerrado (TU); or a control diet + 15% tucum-do-cerrado + AOM (TU/DR). The association of tucum-do-cerrado and AOM (TU/DR) increased glutathione-S-transferase activity, decreased MDA levels, increased levels of COX2, TNFα and BAX, and decreased Bcl2/Bax ratio, compared to the CT/DR group. Carbonyl levels, IL-1β and IL-6 mRNA levels, and aberrant crypt foci showed no difference between the treatments. In conclusion, tucum-do-cerrado reduced lipid oxidative damage, induced a pro-inflammatory effect, and promoted a pro-apoptotic “environment” in rats treated with AOM; however no changes in aberrant crypts were observed.

## Introduction

According to the World Health Organization (WHO, 2015), cancer is the second leading cause of death in the world, accounting for 8.8 million deaths in 2015. The global incidence of the disease is high and tends to grow as the population ages, with 21.4 million new cases expected by 2030. Colon cancer is the second and third most common type of cancer in women and men, respectively. In 2015, colorectal cancer was responsible for 774 thousand deaths [1].

During the development of cancer, cancer cells acquire mechanisms to resist cell death and thus sustain their progression. The mammalian organism has several mechanisms that induce apoptosis, one of them mediated by Bcl2-associated X protein (BAX, pro-apoptotic protein) and B-cell lymphoma 2 (Bcl-2, anti-apoptotic protein) proteins. Bcl-2 acts as a guardian of the mitochondrial membrane, maintaining the integrity of the outer membrane and preventing the release of cytochrome c into the cytosol. On the other hand, BAX promotes the release of cytochrome c, which leads to the activation of caspases and consequently marks the cell to die. In cancer cells changes in the expression of these proteins leading to an anti-apoptotic profile have been well documented [2].

Inflammation and oxidative stress have several effects on cancer cells [3, 4]. Pro-inflammatory cytokines might contribute to tumor development in the three stages of cancer: initiation, promotion, and progression [5]. Ciclooxygenase-2 (COX-2) activity has also been linked to cancer development and the literature shows that COX-2 inhibition results in the prevention of colon cancer growth [6, 7].

Although the relationship between reactive species and carcinogenesis is highly complex, the involvement of reactive species in the three stages of tumor development (initiation, promotion, and progression) is widely described in the literature [3]. The first connection between reactive species and cell transformation was described in the literature in 1981 [8] and to this day the role of reactive species in cancer development remains controversial [9]. Reactive species may exert an oncogenic or tumor-suppressive effect depending on the type of tissue [10], the type, amount, and time of reactive species produced in the tissue, and the tumor stage [3, 11, 12].

Azoxymethane (AOM) treatment is a widely used model that mimic human colon cancer in rodents, and used to study possible chemopreventive effects of foods on colon cancer [13]. After intra-peritoneal injections of AOM, the drug is metabolized in methylazoxymethanol by liver cytochrome P450. After this first step, the MAM goes to the intestine in which it breaks down in formaldehyde and methyldiazonium, a highly reactive alkylating agent that causes a locally alkalinization, via methylation, of guanine and thiamine from DNA [14]. Mutation of K-ras, β-catenin and TGF-β genes caused by methyldiazonium have been described in the literature and it can initiate the process of colon tumorigenesis [13].

Studies show that about one third of all plants and vegetables exhibit antioxidant and anti-inflammatory action, therefore diet plays an important role in both cancer development and prevention [15]. In order to identify whether natural components found in fruits [16], plants [17], and vegetables [18] have a chemo-preventive effect on the development of colon cancer, some *in vitro* and *in vivo* studies have been performed.

Studies conducted by our research group have demonstrated that tucum-do-cerrado, a fruit found in the Brazilian Cerrado and rich in phytochemical compounds, presents high antioxidant activity *in vivo* [19] and *in vitro* [20]. The literature shows that foods rich in different phytochemical compounds modulate inflammation [21], apoptosis [22], and the redox response [11]. Considering that the modulation of these responses by phytochemical compounds may result in anticancer effects, this study aimed to test the chemopreventive effect of tucum-do-cerrado on the development of azoxymethane-induced colon cancer in rats.

## Material and method

### Animals and study design

Newborn (21 days) male Wistar rats were obtained from Granja RG, Santo Amaro, São Paulo, Brazil (n = 28). The animals were housed individually in a room with a 12h dark/light cycle, at a temperature of 23 ± 2°C, with free access to water and food during the dark cycle. The rats were acclimated for 27 days with the standard diet for AIN-93G rodents [23] until they reached the minimum weight (220g) to receive the azoxymethane (AOM). The study was approved by the Animal Care and Use Committee of the University of Brasília, under experimental protocol UnBDoc 160500/2013.

**Fig 1:**
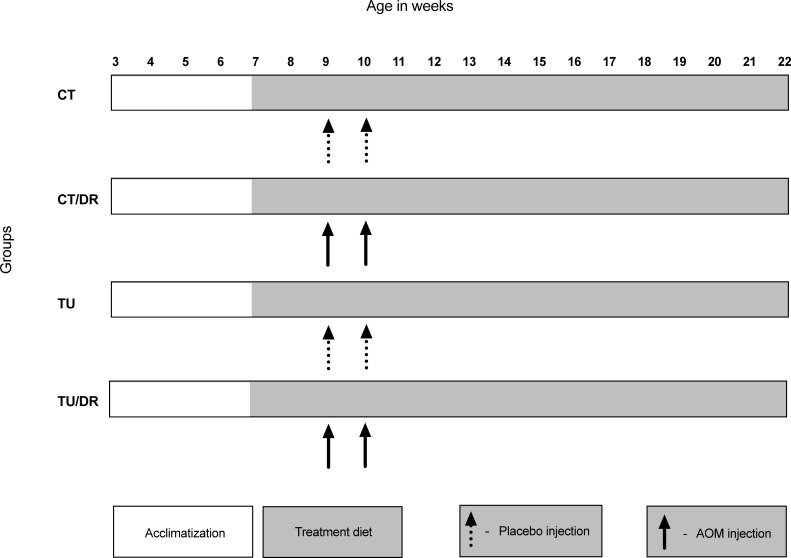
Study design. Group control diet + placebo injection (CT); group control diet + AOM injection (CT/DR); group tucum-do-cerrado diet + placebo injection (TU); group tucum-do-cerrado diet + AOM injection (TU/DR).

After acclimation, the rats were divided into 4 different groups: (i) a control group (CT; n = 6), which was fed with an AIN-93G diet [23] and received saline injections; (ii) a control diet + AOM injection group (CT/DR; n = 8), which was fed with an AIN-93G diet and received AOM injections; (iii) a tucum-do-cerrado group (TU; n = 6), which was fed with an AIN-93G diet containing 150 g of tucum-do-cerrado/kg diet and received saline injections; and (iv) a tucum-do-cerrado diet + AOM injection group (TU/DR; n = 8), which was fed with an AIN-93G diet containing 150 g of tucum-do-cerrado/kg diet and received AOM injections. The diet containing tucum-do-cerrado was made by adding 150g of the whole fresh fruit (pulp and skin) in the control diet discounting the macronutrients presented in tucum-do-cerrado. AOM (Sigma Aldrich, 15 mg/Kg body weight) or saline (saline 0.9%, 15 mg/Kg body weight) was injected two weeks after starting the treatment with the experimental diets, followed by a second injection one week later [24] (Fig 1). Twelve weeks after the second AOM or placebo injection, the rats were anesthetized in an anesthetic chamber with isoflurane and the blood was collected by cardiac puncture. Liver and colon were excised and rinsed with saline. The liver was immediately frozen in liquid nitrogen and the colon was split longitudinally and one half rapidly frozen in liquid nitrogen and the other half kept in 10% neutral buffered formalin for aberrant crypt foci evaluation [25]. The tissues were stored at -80°C until analysis.

### Food intake and weight record

The animals were weighed weekly and diet intake was measured daily by the difference between the amount of food offered and left.

### Hematological parameters

The blood was collected in tubes containing 7.0% EDTA and the analysis of hematological parameters was performed in an ABX Micros ESV60 (Horiba, Kyoto, Japan) cell counter by the Veterinary Hospital of Brasilia University. The following parameters were analyzed: erythrocytes, hemoglobin, hematocrit, mean corpuscular volume (MCV), mean corpuscular hemoglobin (HCM), mean corpuscular hemoglobin concentration (MCHC), platelets, and white blood cells.

### Aberrant crypt foci (ACF)

**Fig 2:**
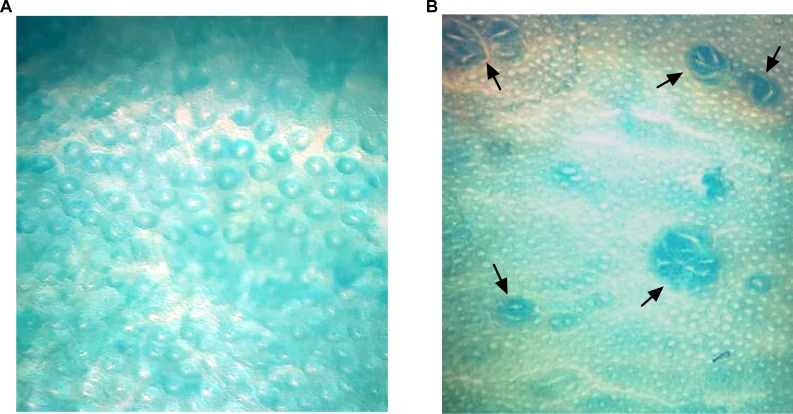
(A) Topographical view of normal crypts (20×). (B) Aberrant crypt foci seen in whole mount of colon stained with methylene blue (40×).

The first change observed in the colon that can result in cancer is the formation of a single preneoplastic cell (aberrant crypts; AC), which can develop into a cluster of preneoplastic lesions known as aberrant crypts foci (ACF). Foci containing four or more aberrant crypts (high multiplicity ACF; HMACF) are predictive of eventual tumor formation [26]. Identification and quantification of ACF in the colon was carried out according to the method described by Bird [27]. The colon kept in 10% formalin was stained with 0.2% methylene blue solution for approximately 1 minute and placed on a microscope with the mucosal surface facing up. The number of AC foci was counted and classified by its multiplicity using a light microscope at x40 magnification. The multiplicity was evaluated by counting the number of aberrant crypts presented in each foci (Fig 2).

### Lipid peroxidation

The levels of malondialdehyde (MDA) in the liver and colon were determined using high-performance liquid chromatography as described by Candan and Tuzmen [28], with some modifications [29]. Concisely, the tissue was homogenized in 1% sulfuric acid with a final dilution of 1:10 (weight/volume) and centrifuged at 12,000 × g for 15 min at 4°C. An aliquot of the supernatant (250 μL) was added to 375 μL of 440 mmol/L phosphoric acid and 125 μL of 42 mmol/L 2-thiobarbituric acid (TBA; Sigma, St. Louis, MO, USA). The mixture was heated for 1 hour at 100°C and, after cooling down, 500 μL of this mixture was added to 500 μL of methanol and 1 mol/L NaOH (91:9 volume/volume). The samples were centrifuged at 12,000 × g for 5 min at 4°C, the supernatant filtered in polyvinylidene difluoride (PVDF) membrane 0.45 μm, and 50 μL of each sample was injected into a HPLC system using a 15 cm Shim-park C18 CLC-ODS (M) column (Shimadzu, Kyoto, Japan). The mobile phase used in the system was metanol:phosphate buffer 50 mmol/L (60:40) and the spectrofluorometric detector wavelengths were set to 553 nm (emission) and 532 nm (excitation). The standard curve was formed by the acid hydrolyses of tetraethoxypropane (TEP; Sigma, St. Louis, MO, USA) in 1% sulfuric acid using four standard points (0.81–16.16 nmol/mL; y = 1^−6^ X + 0.0719; r^2^ = 0.9911). The results were expressed as nmol MDA/mg total protein, and the protein concentration was determined as in the method described before [30].

### Protein oxidation

Carbonyl levels were measured in the liver and colon by a spectrophotometer (Shimadzu–TCC 240A) set at 376 nm, as previously described by Richert, Wehr [31]. The results were expressed in nmol carbonyl/mg total protein using an extinction coefficient of 22.000 mM-1cm-1. The total protein concentration of each sample was analyzed as described by Hartree [30].

### Tissue homogenates for enzyme assays

The activity of the catalase, glutathione peroxidase, glutathione reductase, and glutathione-S-transferase enzymes was determined in the same homogenate. Briefly, the liver (final dilution of 1:40 weight/volume) and the colon (final dilution of 1:20 weight/volume) were homogenized in 50 mmol/L potassium phosphate buffer pH 7.2, containing 0.5 mmol/L ethylenediaminetetra-acetic acid (EDTA) and 1 mmol/L phenylmethylsulfonyl fluoride (PMSF; Sigma Aldrich, St. Louis, MO, USA). Samples were than centrifuged at 13,000 x g for 20 min at 4°C and the supernatant used for all the analyses. A different homogenate was used for the quantification of *s*uperoxide dismutase activity: liver and colon were homogenized in 50 mmol/L potassium phosphate buffer pH 7.8, containing 0.5 mmol/L of EDTA and 1 mmol/L of PMSF, with a final dilution of 1:20 (weight/volume).

#### Catalase activity

The consumption of H_2_O_2_ was measured by a spectrophotometer (Shimadzu–TCC 240A) set at 240 nm [32] for 20 seconds and the catalase activity was determined using the extinction coefficient of H_2_O_2_ (0.0394 mM-1cm-1). The reaction system was composed of 60 μL of tissue homogenates for the colon and 5 μL for the liver, 50 mmol/L potassium phosphate buffer pH 7.2, 0.5 mmol/L EDTA, and 10 mmol/L H_2_O_2_, in the final concentrations. The blank was composed of the reaction system without H_2_O_2_. One unit of catalase was determined as the amount of enzyme required to decompose 1mmol of H_2_O_2_/min.

#### Glutathione Reductase (GR) activity

The assay was performed in accordance with Joanisse and Storey [32]. In this system, nicotinamide adenine dinucleotide phosphate (NADPH; Sigma Aldrich, St. Louis, MO, USA) acts as an electron donor that reduces oxidized glutathione (GSSG; Sigma Aldrich, St. Louis, MO, USA) and its oxidation was monitored using a spectrophotometer (Shimadzu–TCC 240A) set at 340 nm for 30 seconds. The reaction system contained 40 μL of tissue homogenates for both colon and liver, 50 mmol/L potassium phosphate buffer pH 7.2, 0.5 mmol/L EDTA, 0.2 mmol/L NADPH, and GSSG 1 mmol/L, in the final concentrations. The blank was composed of the reaction system without GSSG. GR activity was determined using the extinction coefficient of NADPH (6.22 mM-1cm-1). One unit of GR was defined as the amount of enzyme required to decompose 1 nmol of NADPH/min.

#### Glutathione Peroxidase (GPx) activity

The GPx activity was determined by the oxidation of NADPH in a reaction catalyzed by glutathione reductase (GR) whose substrate is GSSG [32]. The reaction was monitored by a spectrophotometer (Shimadzu–TCC 240A) set at 340 nm for 20 seconds. The reaction system contained 75 μL of tissue homogenates for the colon and 20 μL for the liver, 50 mmol/L potassium phosphate buffer pH 7.2, 0.5 mmol/L EDTA, 2 mmol/L sodium azide (Sigma Aldrich, St. Louis, MO, USA), 1,5 U/mL GR (Sigma Aldrich, St. Louis, MO, USA), 0.15 mmol/L NADPH, 5 mmol/L GSH (Sigma Aldrich, St. Louis, MO, USA), and 0.2 mmol/L H_2_O_2_. The blank was composed of the reaction system without H_2_O_2_. The extinction coefficient of NADPH (6.22 mM-1cm-1) was used to determine GPx activity. One unit of GR was determined as the amount of enzyme required to decompose 1 nmol of NADPH/min.

#### Glutathione-S-transferase (GST) activity

The GST activity was evaluated by monitoring the conjugation of 1-chloro-2,4-dinitrobenzene (CDNB; Sigma Aldrich, St. Louis, MO, USA) with GSH (S-2,4-dinitrophenyl glutathione; Sigma Aldrich, St. Louis, MO, USA) using a spectrophotometer (Shimadzu–TCC 240A) set at 340 nm for 20 seconds. The reaction system was formed of 50 μL of tissue homogenates for colon and liver, 50 mmol/L potassium phosphate buffer pH 7.2, 0.5 mmol/L EDTA, 1 mmol/L CDNB, and 1 mmol/L GSH. The blank was carried out without GSH. One unit of GST was determined as the amount of enzyme required to decompose 1 nmol of conjugate/min [33].

#### Superoxide Dismutase (SOD) activity

In the assay described by [34], xanthine oxidase produces superoxide from the oxidation of hypoxanthine to xanthine, which in turn reduces cytochrome c. The kinetics of the cytochrome c reduction was monitored by a spectrophotometer (Shimadzu–TCC 240A) set at 550 nm for 1 minute. When the homogenate was added to the system, the inhibition of cytochrome c reduction occurs, as SOD catalyzes the dismutation of superoxide into H_2_O_2_ and O_2_. The reaction system, in the final concentrations, was composed of 50 mmol/L potassium phosphate buffer pH 7.2, 0.5 mmol/L EDTA, 0.01 mmol/L oxidized cytochrome c (Sigma Aldrich, St. Louis, MO, USA), 0.05 mmol/L hypoxanthine, homogenate (amount varied from 5 μL to 80 μL), and enough quantity of xanthine oxidase to generate a cytochrome c reduction rate of 0.025 abs/min. One unit of SOD was determined as the amount of enzyme in the homogenate required to decrease the cytochrome c reduction by 50%. To obtain this data, it was necessary to perform the assay using different volumes of the same homogenate to obtain a logarithmic function from the plot of the amount of SOD and the percentage of the inhibition of cytochrome c reduction.

### RNA extraction and reverse transcription-polymerase chain reaction analysis (qRT-PCR)

The RNA from colon was extracted using Trizol reagent (Invitrogen, Carlsbad, CA, USA) and chloroform. The extracted RNA was washed with ethanol and precipitated with sodium acetate. The integrity of the total RNA was analyzed using agarose gel electrophoresis and the 1D LabImage software (Kapelan Bio-Imaging Solutions, Leipzig, Germany). The concentrations of total RNA samples were determined by the absorbance at 260nm in a spectrophotometer (Ultrospec 3000 UV-visible, Pharmacia Biotech, Cambridge, England) and purity was evaluated using the A260 / A280 and A260 / A230 ratios. The cDNA synthesis was performed using the Improm II Reverse Transcription Kit (Promega, Madison, WI, USA) and for each sample a negative control without the reverse transcriptase enzyme was performed. The mRNA concentrations of Bcl2-associated X protein (Bax), B-cell CLL/lymphoma 2 (Bcl2), cyclooxygenase-2 (Cox2), interleukin 6 (Il6), interleukin 1 beta (Il1β), and tumor necrosis factor alpha (Tnfa) were determined using the real time quantitative polymerase chain reaction (q*RT*-PCR) in the 7500 Fast Real-Time PCR System (Applied Biosystems, Foster City, CA, USA). The real time PCR reaction was carried out using a Fast SYBR Green Master Mix 2x reagent (Applied Biosystems, Foster City, CA, USA), 2.0 μL of cDNA, and 0.2 μmol/L (final concentration) of each primer, in a final volume of 10 μL. The oligonucleotide sequences used have been previously described in the literature (Table 1). The gene amplification was performed using 50 cycles under the following conditions: at 95° C for 20 seconds, 95° C for 3 seconds, and 60°C 30 seconds. The amplification specificity of each amplicon was verified using a melting curve. The efficiency of the PCR amplification reaction was evaluated using a standard curve constructed from different cDNA dilutions for each gene analyzed. The curve was set up by correlating the ΔCT (threshold cycle) versus the log of the amount of cDNA used for each point on the curve. The amplification efficiency was determined from the slope obtained by the standard curve, and a slope value of less than 0.1 was required. Expression of all genes was normalized using the constitutive β-actin gene (Actb). Thus, the quantification of mRNA levels of the target genes was calculated using the CT of the target gene relative to the constitutive gene (β-actin), by means of the formula 2^-ΔΔCT^. The method described above follows the tutorial from Applied Biosystems (2008).

**Table 1:** Sequences of the oligonucleotides used in the qPCR reaction.

Gene name	Gene		Oligonucleotideo (5'–3')	Reference
Cyclooxygenase 2	Cox2	**F** **R**	GCAAAGGCCTCCATTGACCAGAG CGGGATACAGTTCCATGGCATCG	[35]
B-cell lymphoma 2	Bcl2	**F** **R**	GATTGTGGCCTTCTTTGAGT ATAGTTCCACAAAGGCATCC	[36]
Bcl-2-associated X protein	Bax	**F** **R**	TGCTACAGGGTTTCATCCAG ATCCTCTGCAGCTCCATGTT	[36]
Interleukin 6	Il6	**F** **R**	GCCAGAGTCATTCAGAGCAATA GTTGGATGGTCTTGGTCCTTAG	[37]
Interleukin 1 beta	Il1β	**F** **R**	CACCTCTCAAGCAGAGCACAG GGGTTCCATGGTGAAGTCAAC	[38]
Tumor necrosis fator alpha	Tnfa	**F** **R**	AAATGGGCTCCCTCTCATCAGTTC GTCGTAGCAAACCACCAAGCAGA	[38]
β actin	Actb	**F** **R**	GTCGTACCACTGGCATTGTG CTCTCAGCTGTGGTGGTGAA	[39]

### Determination of Bcl2-associated X protein, B-cell lymphoma 2, and cyclooxygenase-2 protein levels via immunoblot analysis

An aliquot of the colon was homogenized in buffer (0.1g : 200 μL) containing 0.25 mol/L sucrose, 15 mmol/L Tris HCl pH 7.9, 15 mmol/L NaCl, 60 mmol/L KCl, 5 mmol/L EDTA, 0.15 mmol/L Spermine, 0.5 mmol/L Spermidine, 1 mmol/L Dithiotheritol, 1% inhibitor cocktail (volume/volume buffer; Sigma Aldrich St. Louis, USA), and 1% phosphatase inhibitor cocktail (volume / volume buffer; Sigma Aldrich, St. Louis, USA) using an ice-submerged glass-glass homogenizer. The sample was then sonicated for 1 minute, centrifuged at 10,000 x g for 10 minutes, and the supernatant was collected. An aliquot of the supernatant was used to determine the total protein concentration by means of the method described by Hartree [30] and the remainder was stored at -80°C. Samples for polyacrylamide gel electrophoresis were prepared by diluting homogenate in water in order to load a total protein amount of 45 μg for COX-2 and 20 μg for Bcl-2 and BAX in each well. The diluted samples were then added to a buffer containing 1.2 mol/L Tris HCl pH 6.8, 4% SDS weight/volume, 20% glycerol (volume/volume), and 0.1% bromophenol blue in a proportion of 1:1. The β-mercaptoethanol (60:1; Sigma Aldrich St. Louis, USA) was added to the sample and the solution heated at 100°C for 1 minute. The proteins were separated in a 12% SDS-PAGE.

After gel separation, the proteins were transferred to a 0.45 μm polyvinylidene difloride membrane (PVDF—ImmobilonH-P transfer membrane—IPVH00010—Millipore—Billerica, Massachusetts, USA) using a Semi-Dry Trans Blot system (Trans-Blot SD Semi-Dry Transfer Cell, BIO-RAD, Hercules, CA, USA) with extra thick filter paper moistened with 48 mmol/L tris, 39 mmol/L glycine, and 20% methanol. The transfer conditions varied according to the protein of interest: 15 V for 30 minutes for Bcl-2 and BAX and 15 V for 40 minutes for COX-2. The membrane was then blocked with 5% skimmed milk powder in TBST (20 mmol/L tris, 0.137 mol/L NaCl, 0.1% tween 20) for 2 hours in a shaker at room temperature. The membrane was washed twice with TBST solution and incubated with a primary antibody diluted in blocking solution (1:2000 for β-actin, 1:500 for COX-2, 1:1000 for BAX and Bcl-2) for 17 hours in a shaker at 5°C. Subsequently, the membrane was washed three times with TBST and incubated with a secondary antibody (1:1000) in a shaker at room temperature for 1 hour. The membrane was re-washed with TBST three times and the bands were visualized with BCIP / NBT solution (Sigma Aldrich, St. Louis, USA) after incubation for 30 minutes (BAX and BCL-2) or 6 hours (COX-2). The bands were quantified in the Image Studio image analysis system (Li-cor-Bioscience, Lincon, NE, USA).

### Determination of levels of tumor necrosis factor-alpha (TNFα), interleukin-6 (IL-6), and interleukin 1β (IL-1β)

Commercial immunoenzymatic assay kits (ELISA) were used to determine the levels of IL-6 (Thermo Fisher Scientific; Massachusetts, USA), TNFα, and IL-1β (eBioscience, Vienna, Austria) in serum. The analyses were performed following the manufacturer's recommendations.

### Statistical analyses

The normality of the data distribution was analyzed using the Kolmogorov-Simirnov test. Differences between the treatment groups were analyzed using the One-Way ANOVA test with Bonferroni’s post-tests for normal distribution variables, and for non-normal distribution variables the Kruskal-Wallis test was performed. The significance level established for all tests was P ≤ 0.05. All the analyses were performed using the SPSS 19.0 software (SPSS Inc., Chicago, IL).

## Results

### Food intake, weight gain, and hematological parameters

Table 2 shows the food intake and weight gain of the rats during the twelve weeks of treatment. Neither the AOM injections nor tucum-do-cerrado consumption altered food intake and weight gain among the groups.

**Table 2:** Effect of tucum-do-cerrado consumption in food intake, weight gain and hematological parameters in rats treated with AOM and placebo.

Groups				
	CT	CT/DR	TU	TU/DR
**Food intake (Kg)**	2.64 ± 0.21	2.71 ± 0.19	2.68 ± 0.09	2.78 ± 0.12
**Weight gain (g)**	249.4 ± 47.3	268.1 ± 13.9	236.3 ± 12.2	253.3 ± 46.0
**Red blood cells (million/mm**^**3**^**)**	7.77 ± 0.45	7.65 ± 0.51	7.79 ± 0.43	7.82 ± 0.60
**Hematocrit (%)**	44.24 ± 2.36	42.6 ± 3.74	41.92 ± 1.56	43.42 ± 2.85
**Hemoglobin (g/dL)**	15.40 ± 0.77	15.50 ± 0.66	15.02 ± 0.68	15.46 ± 0.74
**MCV (fl)**	56.91 ± 1.46	55.83 ± 1.21	55.00 ± 1.06	55.75 ± 1.71
**MCH (pg)**	20.09 ± 0.18	20.38 ± 0.85	18.98 ± 0.42	19.81 ± 0.96
**MCHC g/dl**	34.83 ± 0.69	36.54 ± 1.92	35.12 ± 0.94	35.58 ± 1.37
**Platelets (mil/mm**^**3**^**)**	542 ± 33	529 ± 54	542 ± 59	537 ± 96
**Leukocytes (/mm**^**3**^**)**	3.55 ± 0.50	5.43 ± 0.98*	4.51 ± 0.81	4.33 ± 1.16

CT, group treated with control diet AIN-93G + placebo injection; CT/DR, group treated with AIN-93G diet + AOM injection; TU, group treated with AIN-93G diet added of 15% tucum-do-cerrado + placebo injection; TU/DR, group treated AIN-93G diet added of 15% tucum-do-cerrado + AOM injection. Data are the mean ± standard deviation (n = 25).

*Different than CT group

^$^different than CT DR group (p ≤ 0.05).

For the hematological parameters, the AOM treatment (CT/DR group) increased leukocyte levels compared with the control group (Table 2). The consumption of tucum-do-cerrado associated or not with AOM treatment did not alter the evaluated hematological parameters.

### Incidence of aberrant crypt foci (ACF) in the colon

The AOM injections in the CT/DR and TU/DR groups induced the development of aberrant crypts (AC), aberrant crypt foci (ACF), and high multiplicity aberrant crypt foci (HMACF; foci > 4 AC / focus) in the whole colon. However, the consumption of tucum-do-cerrado associated to AOM injections (TU/DR group) did not alter AOM-induced ACF formation in the colon compared with the CT/DR group (Table 3). The CT and TU groups did not show aberrant crypts in the colon.

**Table 3:** Effect of tucum-do-cerrado consumption in the number of AC/cm, ACF/cm and HMACF/cm in the colon of rats treated with AOM.

.Groups	AC / cm^.^	ACF / cm^.^	HMACF / cm^.^
**CT/DR**	14.65 ± 3.64	7.46 ± 1.22	0.969 ± 0.296
**TU/DR**	16.50 ± 4.15	7.86 ± 2.29	1.092 ± 0.313

AC: aberrant crypt; ACF: aberrant crypt foci; HMACF: high multiplicity aberrant crypt foci (foci > 4 AC / focus). CT/DR, group treated with AIN-93G diet + AOM injection; TU/DR, group treated AIN-93G diet added of 15% tucum-do-cerrado + AOM injection. No aberrant crypts were detected in the CT and TU groups. Data are the mean ± standard deviation (n = 14). *Different than CT group; ^$^different than CT DR group (p ≤ 0.05).

### Oxidative damage and antioxidant enzyme activity

**Fig 3:**
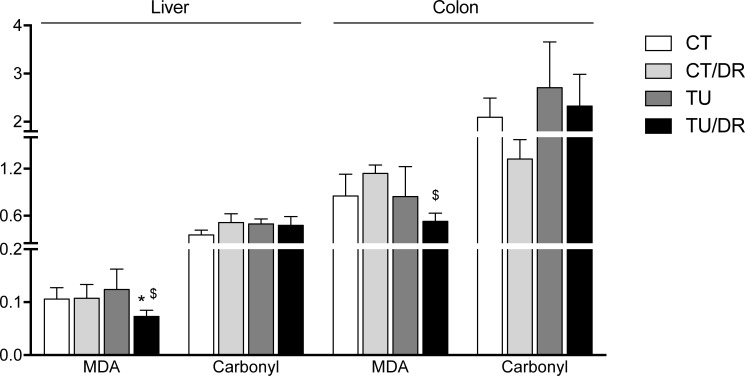
Effect of tucum-do-cerrado consumption in lipid oxidation (MDA) and protein (carbonyl) in liver and colon from rats treated with AOM and placebo. CT, group treated with control diet AIN-93G + placebo injection; CT/DR, group treated with AIN-93G diet + AOM injection; TU, group treated with AIN-93G diet added of 15% tucum-do-cerrado + placebo injection; TU/DR, group treated AIN-93G diet added of 15% tucum-do-cerrado + AOM injection. Data are the means ± standard deviation (n = 23). *Different than CT group; ^$^different than CT DR group (p ≤ 0.05).

Although the AOM treatment (CT/DR) did not change the hepatic MDA levels, the association of tucum-do-cerrado diet with AOM injection (TU/DR) reduced the levels of MDA in the liver and colon of these rats, compared with the rats treated with the control diet + AOM injection (CT/DR; Fig 3). In the liver, the association of the tucum-do-cerrado diet with AOM injection (TU/DR) decreased the levels of MDA even compared with the control group (CT). No differences were found in carbonyl levels in the liver and colon in any of the treatment groups (Fig 3).

**Fig 4:**
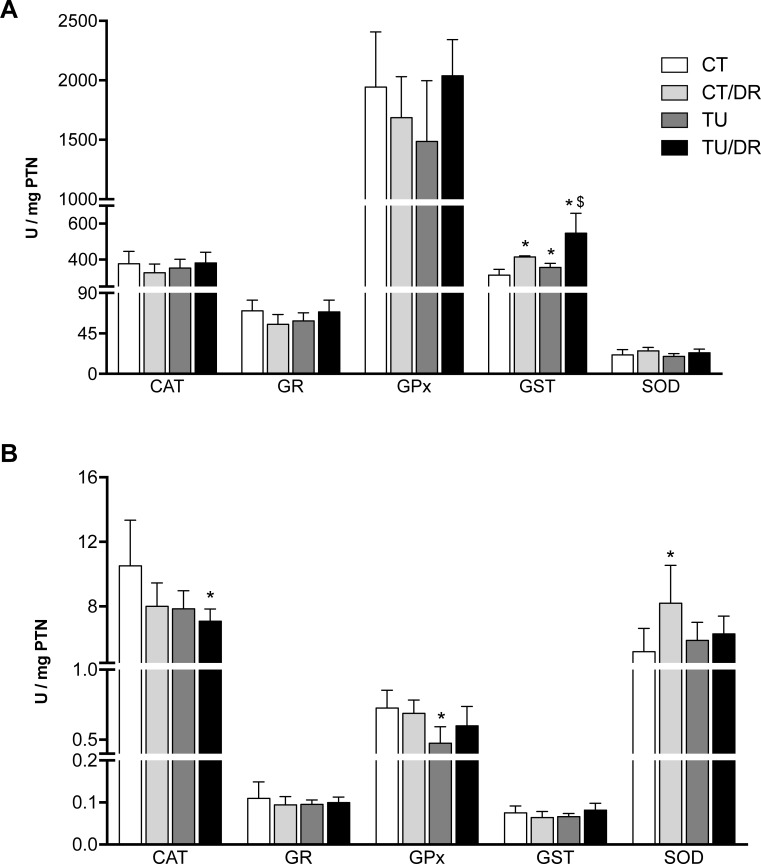
**Effect of tucum-do-cerrado consumption in antioxidant enzyme activity in the liver (A) and colon (B) of rats treated with AOM and placebo.** CAT: catalase; GR: glutathione reductase; GPx: glutathione peroxidase; GST: glutathione-S-transferase; SOD: superoxide dismutase. CT, group treated with control diet AIN-93G + placebo injection; CT/DR, group treated with AIN-93G diet + AOM injection; TU, group treated with AIN-93G diet added of 15% tucum-do-cerrado + placebo injection; TU/DR, group treated AIN-93G diet added of 15% tucum-do-cerrado + AOM injection. Data are the means ± standard deviation (n = 23). *Different than CT group; ^$^different than CT DR group (p ≤ 0.05).

Regarding the antioxidant response, AOM injection (CT/DR) and tucum-do-cerrado consumption (TU), as well as the association of both treatments, tucum-do-cerrado consumption + AOM injection (TU/DR), increased hepatic GST activity compared with the control group (CT). The group treated with the association of tucum-do-cerrado diet and AOM injection (TU/DR) also showed higher GST activity in the liver compared with the control diet + AOM injection group (CT/DR). No difference was obtained for catalase, glutathione reductase, glutathione peroxidase, and superoxide dismutase specific activities in the liver in any of the treatments (Fig 4A).

In the colon, the association of the tucum-do-cerrado diet with the AOM injection (TU/DR) decreased catalase activity compared with the control group. Furthermore, the tucum-do-cerrado diet (TU) decreased GPx activity and the injection of AOM (CT/DR) increased SOD activity compared with the control group (Fig 4B). The specific activity of GR and GST in the colon did not alter with the AOM injection (CT/DR), tucum-do-cerrado consumption (TU), or the association of both treatments (TU/DR).

### Expression of apoptosis-associated proteins: Bcl2-associated X protein (BAX) and B-cell CLL/lymphoma 2 (Bcl-2)

**Fig 5:**
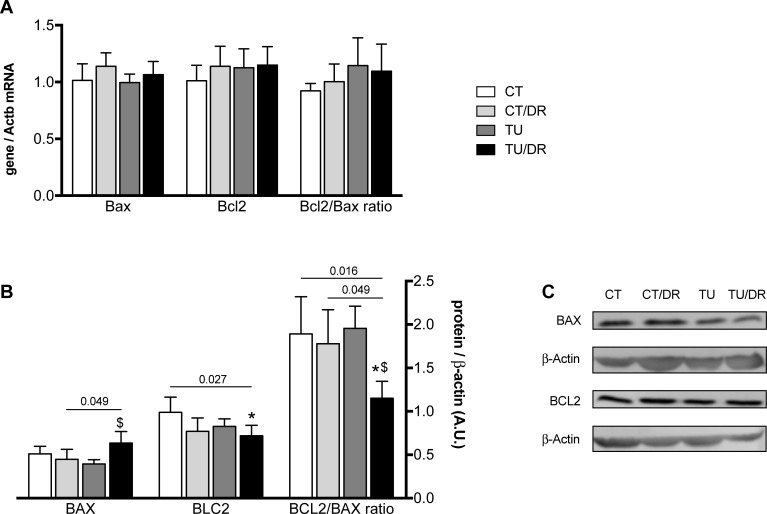
**Effect of tucum-do-cerrado consumption in the mRNA (A) and protein (B, C) levels of apoptosis related proteins, BAX and Bcl-2, in colon of rats treated with AOM and placebo.** CT, group treated with control diet AIN-93G + placebo injection; CT/DR, group treated with AIN-93G diet + AOM injection; TU, group treated with AIN-93G diet added of 15% tucum-do-cerrado + placebo injection; TU/DR, group treated AIN-93G diet added of 15% tucum-do-cerrado + AOM injection. Data are the means ± standard deviation (n = 23). *Different than CT group; ^$^different than CT DR group (p ≤ 0.05).

The mRNA and protein levels of BAX and Bcl-2 and the Bcl-2/BAX ratio were evaluated in the colon as apoptosis biomarkers [40]. No differences were obtained in the mRNA levels of Bax or Bcl2 as well as in the Bcl2/Bax mRNA ratio in any of the treatment groups. Although tucum-do-cerrado consumption (TU) and AOM treatment (CT/DR) did not modulate BAX and Bcl-2 protein levels, the association of tucum-do-cerrado diet with AOM injection (TU/DR) promoted an increase in BAX protein levels and consequently a decrease in the Bcl-2/BAX protein ratio in the colon of rats, compared with the group treated only with AOM (CT/DR; Fig 5). A decrease was also observed in the Bcl-2 protein level and consequently in the Bcl-2/BAX protein ratio, when the tucum-do-cerrado diet was associated with the AOM injection (TU/DR), compared with the control group.

### Cytokine expression

**Fig 6:**
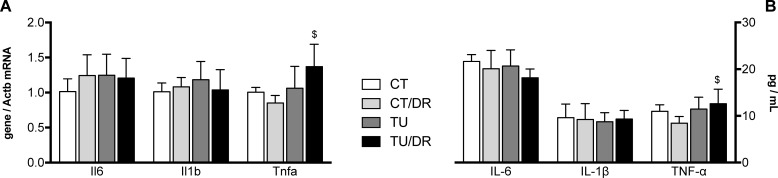
**Effect of tucum-do-cerrado consumption in the colon levels of cytokines mRNA (A) and serum protein (B) of rats treated with AOM and placebo.** CT, group treated with control diet AIN-93G + placebo injection; CT/DR, group treated with AIN-93G diet + AOM injection; TU, group treated with AIN-93G diet added of 15% tucum-do-cerrado + placebo injection; TU/DR, group treated AIN-93G diet added of 15% tucum-do-cerrado + AOM injection. Data are the means ± standard deviation (n = 25). *Different than CT group; ^$^different than CT DR group (p ≤ 0.05).

Fig 6 shows the mRNA and protein levels of proinflammatory cytokines IL-6, IL-1β, and TNFα in the serum. The TNFα mRNA and protein levels were upregulated in the group treated with the association of tucum-do-cerrado diet with AOM injection (TU/DR) compared with the group treated with the control diet + AOM injection (CT/DR), even though tucum-do-cerrado consumption (TU) and AOM injection (CT/DR) did not alter this biomaker. No differences were obtained in protein or mRNA levels of IL-6 and IL-1β in any of the treatments.

### Cyclooxygenase 2 expression

**Fig 7:**
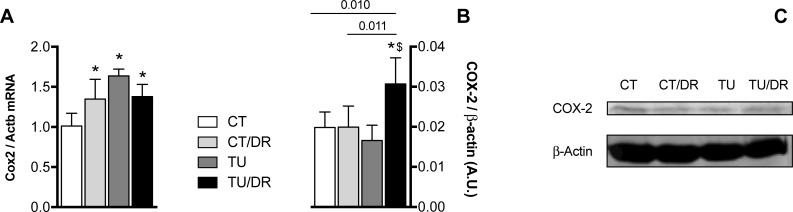
**Effect of tucum-do-cerrado consumption in the mRNA (A) and protein (B, C) levels of cyclooxygenase 2 in colon of rats treated with AOM and placebo.** CT, group treated with control diet AIN-93G + placebo injection; CT/DR, group treated with AIN-93G diet + AOM injection; TU, group treated with AIN-93G diet added of 15% tucum-do-cerrado + placebo injection; TU/DR, group treated AIN-93G diet added of 15% tucum-do-cerrado + AOM injection. Data are the means ± standard deviation (n = 23). *Different than CT group; ^$^different than CT DR group (p ≤ 0.05).

The cyclooxygenase 2 mRNA and protein levels in the colon are shown in Fig 7. The mRNA levels of Cox2 were upregulated in the groups treated with tucum-do-cerrado (TU), the control diet + AOM injection (CT/DR), and the association of tucum-do-cerrado diet with AOM injection (TU/DR), compared with the control group (CT). However, only the group treated with the tucum-do-cerrado diet associated with the AOM injection (TU/DR) showed higher protein levels of COX-2 compared to the AOM-treated group (CT/DR).

## Discussion & conclusion

Tucum-do-cerrado is a native Brazilian fruit with a high phenolic compound content [41], therefore in this study we evaluated whether tucum-do-cerrado consumption has chemopreventive effects on the development of AOM-induced colon cancer in Wistar rats using indirect read outs markers for oxidative stress, inflammation and apoptosis. The tucum-do-cerrado ingestion by rats treated with AOM induced a decrease in levels of MDA and in an increase in levels of pro-inflammatory biomarkers, TNF-α and COX-2, and in levels of pro-apoptotic protein BAX.

Azoxymethane (AOM) is a highly carcinogenic drug used to induce colon cancer in rats and mice [13]. After intraperitoneal or subcutaneous injection, AOM is metabolized by cytochrome P450 in the liver, giving rise to carcinogenic metabolites, DNA adducts [42], and oxidative stress [43]. GST forms part of the phase II detoxification enzymes which catalyze the conjugation reaction between different xenobiotics and GSH peptides [44]. In this study, the higher hepatic GST activity observed in the AOM-treated rats (CT/DR) compared with the control rats might be explained by an attempt by the liver to eliminate reactive intermediates of AOM metabolism [45]. Similarly to what was observed in the CT/DR group, tucum-do-cerrado consumption also increased hepatic GST activity. In agreement with our result, other studies have shown that phytochemical compounds induce phase II metabolizing enzymes such as GST, enhancing liver detoxification capacity [44, 46, 47]. An additive effect was observed when tucum-do-cerrado was associated with AOM treatment (TU/DR), once hepatic GST specific activity of TU/DR group was higher than CT/DR, suggesting an attempt of tucum-do-cerrado phytochemicals to eliminate the mutagenic compounds produced by AOM metabolization, to decrease oxidative damage and consequently help in cancer prevention [45]. Considering that the SOD enzyme catalyzes the dismutation of O_2_^-^ into H_2_O_2_ [34], the higher SOD activity obtained in the colon of AOM-treated rats (CT/DR) demonstrated that AOM induces oxidative stress. Although the association of tucum-do-cerrado with AOM (TU/DR) did not alter SOD activity in the colon compared to the CT/DR treatment, it was similar to the control group, suggesting that tucum-do-cerrado compounds were able to neutralize free radicals produced by AOM injection. Phytochemical compounds can exert antioxidant actions and reduce oxidative stress through different mechanisms such as up-regulating antioxidant enzymes [48], chelating metals that are a source of free electrons, and neutralizing free radicals via a direct radical-scavenging ability [3]. In this study, tucum-do-cerrado decreased GPx activity in the colon suggesting that the high content of antioxidant compounds present in tucum-do-cerrado may have diminished the oxidative status of the colon, lowering the need for endogenous production of antioxidant enzymes.

The lower levels of lipid oxidation (MDA) observed in the colon and liver of rats treated with tucum-do-cerrado associated with AOM (TU/DR) compared to the AOM treatment (CT/DR) may be related to the increased hepatic GST activity and to the high antioxidant capacity of tucum-do-cerrado, as previously described by Rosa, Arruda [41].

In order to investigate the effect of tucum-do-cerrado consumption on the expression of apoptosis related factors, BAX and Bcl-2 mRNA and protein levels were evaluated. Intrinsic apoptosis pathway homeostasis is maintained by a balance of anti- (Bcl-2) and pro-apoptotic (BAX) protein levels [49], and it has been suggested that the cellular Bcl-2/BAX ratio is a determinant for the downstream effect of these proteins [40]. In cancer cells, apoptosis is usually disrupted, favoring cell death resistance [2]. Kauntz, Bousserouel [22] showed that the phytochemical silibinin activates apoptosis by upregulating BAX and downregulating Bcl-2 protein and gene expression in rats treated with AOM. In this study, the association of tucum-do-cerrado diet with AOM injection induced an increase in BAX protein levels in the colon and a consequent decrease in the Bcl-2/BAX protein ratio, suggesting that tucum-do-cerrado in the presence of a carcinogenic drug may trigger the intrinsic apoptosis pathway by inducing a pro-apoptotic response. However, no difference was observed in the numbers of aberrant crypts, aberrant crypts foci, and its multiplicity between the TU/DR and CT/DR groups. The modulation of apoptosis related proteins by tucum-do-cerrado consumption in the presence of AOM seems to be a post-transcriptional regulation, as colon Bax and Bcl2 mRNA levels were similar in all the groups. Different post-transcriptional and post-translational regulation of Bcl-2 family proteins by phytochemical compounds have already been reported in the literature [50].

The role of immune response in pre-malignant and malignant tissue is very complex. The activation of the inflammatory process occurs in response to some injury and may have therapeutic effects if maintained for a short period. However, when the inflammatory response becomes chronic, it induces several alterations in the tissues [51], which may result in enhanced cell proliferation, tumor growth and angiogenesis, tissue invasion, and metastasis [52–55]. Studies that evaluate the effect of phytochemical compounds on the inflammatory response usually report a decrease in the levels of several inflammatory biomarkers. Umesalma and Sudhandiran [56] showed that rats treated with elagic acid showed no differences in COX-2, TNF-α, and IL-6 protein levels compared to a control group; however when elagic acid was associated with a drug that induces colon cancer, the levels of these inflammatory biomarkers decreased compared to a group treated with only the drug. Similar results were observed in 5% açaí-treated mice with induced colitis/colon cancer, which showed lower levels of TNF-α, IL-1β, and IL-6 in relation to the control group, while placebo 5% açaí-treated mice did not show any difference in these cytokines compared to placebo mice [57]. Contrary to what was observed by the studies described above, in this study the association of a colon cancer inducer (AOM) with tucum-do-cerrado promoted an increase in pro-inflammatory biomarkers, serum TNFα, and colon COX-2 protein levels, compared to the AOM-treated group (CT/DR). In addition, the AOM injection (CT/DR) did not alter the colon mRNA levels and serum protein content of IL-6, IL-1β, and TNFα, and colon COX-2 protein levels, compared to the control group. Similar results were observed in the aorta of 12-month-old rats treated with catechin for 9 months, which showed an increase in Cox2 mRNA levels compared to non-treated rats; however a 3 month treatment with catechin reduced Cox2 mRNA levels [58]. The authors suggested that the reducing environment promoted by the long-term exposure to catechin disrupted redox homeostasis leading to Cox2 upregulation. In another study, it was observed that the treatment of human cardiac cells with resveratrol increased TNFα levels and this increment was even higher when the cells were treated with resveratrol associated to TNFα [59]. The authors suggested that resveratrol increases TNFα by activating nuclear factor-κB (NFκB).

Nuclear factor kappa B (NFκB) is a transcription factor that plays a key role in the inflammatory response [60]. Studies show that phytochemicals are able to modulate NFκB levels in many different conditions. Paur, Austenaa [61] showed that some dietary plant extracts as well as some isolated phytochemicals may induce basal NFκB activity and inhibit LPS/TNF-α-induced NFκB activity in monocytes. In another study, it was observed that monocytes preincubated with resveratrol and stimulated with LPS enhance pro-inflammatory cytokine (TNFα, IL-6, and IL-1β) expression by upregulating NFκB [62]. Therefore, in this study, the phytochemicals present in the tucum-do-cerrado fruit may have promoted an upregulation of NFκB activity in the rats with chemically-induced colon cancer and consequently increased TNFα and COX-2 mRNA and protein levels in the TU/DR group compared to the CT/DR group. However, increased NFκB activity should also have stimulated IL-6 and IL-1β expression, but in this study, none of these cytokines showed any differences in the groups. These apparently contradictory results may be associated to the various polyphenol classes found in the tucum-do-cerrado fruit, which may modulate the expression of pro-inflammatory cytokines in a distinct manner. Palomer, Capdevila-Busquets [59] suggested that resveratrol has a dual effect on inflammatory gene expression, since it increased NFκB activity and TNFα levels but decreased IL-6 levels in human cardiac cells. In addition, Romier, Van De Walle [63] found that in human intestinal Caco-2 cells genistein activates NFκB while it inhibits IL-8, a NFκB-regulated cytokine. The authors suggested that genistein may modulate IL-8 production through a NFκB-independent pathway.

In addition, Wang and Mazza [64] investigated the effect of different phenolic compound classes on the production of TNFα in activated macrophages and observed that chlorogenic acid had no effect, quercetin and genistein inhibited, while kaempferol, myricetin, anthocyanidins/anthocyanins, and high anthocyanin extracts induced TNFα production. The authors also demonstrated that the glycosylation of the genistein and daidzein isoflavones modified the previously observed effect of genistein, leading to an increase in TNFα production, while it did not affect daidzein action. Therefore, the high TNFα levels observed in the rats treated with AOM and tucum-do-cerrado (TU/DR) might be associated with the high anthocyanin content present in tucum-do-cerrado peels [41]. An increase in serum TNFα levels was also observed in ten healthy subjects 24 h after receiving a 5g oral dose of resveratrol [62]. In this same study, peripheral blood mononuclear cells pre-incubated with resveratrol and stimulated with LPS showed an increase in TNFα levels and a decrease in the anti-inflammatory cytokine IL-10. The authors suggested that the anti-carcinogenic effect of resveratrol might be mediated by the increase in immune-surveillance, as resveratrol augments the pro-inflammatory profile. It has been suggested that in the cancer immunosurveillance process, the inflammatory response leads to higher levels of immune cells that recognize and eliminate pre-cancerous and cancerous cells [4]. Although in this study the association of tucum-do-cerrado with AOM injection did not increase the total leukocyte count, it is possible that tucum-do-cerrado increased the activity and type of immune cells more associated with cancer immunity, which would benefit cancer immunosurveillance, as described by Falchetti, Fuggetta [65].

One limitation of this study is the short treatment period and consequently the lack of data on the incidence of colon cancer in the control and TU/DR groups.

In conclusion, this study showed that tucum-do-cerrado consumption diminished lipid oxidative damage, induced a pro-inflammatory effect, and promoted a pro-apoptotic “environment” in rats with premalignant lesions in the colon induced with AOM; however no changes in aberrant crypts were observed.
